# Occupational injury proneness in Indian women: A survey in fish processing industries

**DOI:** 10.1186/1745-6673-1-23

**Published:** 2006-09-12

**Authors:** Asim Saha, Anjali Nag, Pranab Kumar Nag

**Affiliations:** 1 National Institute of Occupational Health, Ahmedabad, India; 2 Senior Research Officer (Medical), Occupational Medicine Division, National Institute of Occupational Health, Meghani Nagar, Ahmedabad-380 016, Gujarat, India

## Abstract

A cross sectional survey was initiated to understand the frequency of occupational injury occurrence and the associated factors in the fish processing industries of western India involving 185 randomly selected women subjects. All the subjects were interviewed with the help of an interviewer-administered questionnaire to collect information regarding their personal, occupational and work related morbidity details (including details of occupational injuries). Logistic regression method was used to analyze the data in order to obtain the contribution of individual factors on occupational injuries. This study has shown that work related morbidity like blanching of hand (OR; 2.30, 95%CI; 1.12–4.74) and nature of job like grading (OR; 3.99, 95%CI; 1.41–11.27) and packing (OR; 5.68, 95%CI; 1.65–19.57) had a significant impact on injury causation. This study eventually concludes that apart from nature of job of fish processing workers occupational hazards prevailing in the work environment contribute significantly to the occurrence of work related injuries and prevention of such occupational hazards may help in protecting workers from occupational injuries also.

## Background

Occupational injuries represent a major problem in public health. Severe consequences also do occur as after-effect [1]. Social and economic loss takes place [2,3]. Every year almost one thousand workers die and one fourth of a million are injured in industries in India in organized sectors only. Thousands of others are crippled due to occupational injuries in unorganized sectors. Number of insured persons in the pay roll of permanent disablement benefit reached up to 113,500 with addition of about 15,000 fresh cases of disablement due to employment injury during a single year in India only [4]. So far as the causation of such injuries is concerned, a variety of factors have been found to be responsible for occupational accidents, either directly or indirectly. Work conditions [5], age [6,7], safety training [8], experience [9] and weather [10] have all been designated as responsible factors. Some authors have also shown that the type of employment of the worker (temporary or permanent) [11] is also an important factor in the causation of occupational accidents. In recent times, contribution of poor work environmental conditions [12,13], poor perception of work conditions [12] and presence of disease of adverse health condition in workers [14,15] on occupational injury occurrence has been highlighted. Nature of workplaces being varied, determinants of occupational injury causation has also been different and identification of such responsible factors in relation to a specific work environment has not only helped in exploring the aetiology but also been useful in planning prevention.

In fish processing industry the workers use small sharp knife and their hands come in contact with different sharp body parts of fishes. Use of such small hand tools and manual work often exposes workers to frequent minor injuries [16], which in long term may be harmful. A study conducted in UK [17] estimated that almost one in 10 workers in such profession attend casualty in the course of a year for a work related injury. A postulation of seventeen-knife laceration per 1000 per annum was also made. In addition, low temperature of work environment and frequent contact with ice cold chlorinated water makes the workers suffer from many other morbidities including frequent respiratory irritation (frequent sneezing and/or coughing) at work, headache, blanching of hand etc. Though respiratory [18] and musculoskeletal [19] problems of fish processing workers have been addressed repeatedly, hardly any effort is made to explore the problem of injury occurrence in such workplaces. In this backdrop this study was undertaken not only to understand the frequency of injury occurrence and the associated factors but also to test the hypothesis whether sufferings of workers (due to work or work environment) have any significant contribution to the occurrence of occupational injuries.

## Materials and methods

This cross sectional survey was conducted in the sea fish processing industries situated in Gujarat state of western India. This part of India has number of such industries that employ 20000 women workers. A peculiar feature of these industries is that they are employing women workers only for the job of fish processing (only a few male workers are employed for supportive activities). This was the reason of restricting this study to women workers only. On arrival of fish, grading (including debridement) is done initially to segregate them into different categories. Afterwards peeling is done where necessary. Some fishes are chopped into rings (ring cutting) and some are sent for packing per se. Finally packing is done. Peeling is done mostly manually (needed in case of small shrimps only) and ring cutting is a mechanized process in most of the units. Small hand tools like knife, needle are used in grading and packing. The whole activity is done at a low room temperature and hardly any personal protective equipment being used the hands of workers come in frequent contact with ice and ice cold water. To calculate the sample size for this study, prevalence of the outcome variable in reference group and relative risk of the vulnerable worker group was predicted to be 30% and 1.75% respectively. Thus, the minimum sample size for 5% level of significance and 80% power of study was calculated as 166. We set our target as 200 persons. At first five industries were selected randomly from a list of all the industries of that area. Afterwards, random selection of subjects was done from the list of workers of those five industries by using random numbers generated from Microsoft Excel software. Among the 200 workers, who were approached for study, 185 subjects participated. All of them were interviewed with the help of an interviewer-administered questionnaire to collect information regarding their personal, occupational and work related morbidity details (including details of occupational injuries). Necessary approval for this study was obtained from the institutional ethical committee as well as the scientific advisory committee of National Institute of Occupational Health, India.

Initially, a descriptive analysis was done to observe the personal and occupational characteristics of the study subjects as well as to understand the prevalence of different work related morbidities including occupational injuries. A worker having injury as frequently as once a month (30 days) over the period of last one year was considered as "frequent injury" receiver. Hand injuries being exclusively common in such workplaces this questionnaire survey was restricted to information related to hand injuries only. Moreover, injuries those compelled the workers to be away from the work for at least one shift (loss of wages for a day) were considered for this study. Afterwards, analysis was done with the help of SPSS Release 6.1.4 software to obtain the contribution of different factors on occupational injury occurrence. In univariate analysis the contribution of the variables like age group, job duration group, marital status, education level, nature of job, blanching of hand at work, recurrent musculoskeletal pain, headache during work, recurrent sneezing/coughing (respiratory irritation) at work on injury occurrence was examined. In multivariate analysis, logistic regression method was used to obtain the contribution of individual factors on occupational injuries irrespective of the effect of the other factors. Variables like blanching of fingers due to cold during work (yes, no), education level (illiterate, educated), department/nature of job (department with lowest injury was treated as reference and the risk of others were calculated), marital status (married, unmarried), musculoskeletal problem anywhere in the body (yes, no) and pain in upper limb (yes, no) were taken as categorical variables. Other variables like age (yrs), experience in this job (yrs) were taken as continuous variables. While analyzing, we used three logistic regression models. In the first, we accommodated only three variables (age, education level, marital status). In the second we added the morbidity variables (blanching of hand, musculoskeletal pain, pain in upper limb) also. Finally in the third we added the variables related to work (department/nature of job and experience in the job) and analyzed all variables simultaneously in the model in order to estimate the effect of every individual variable adjusting for the effect of other variables.

## Results

Mean age of the study subjects was 24.4 (± 7.4) years. One hundred & thirty two (71.4%) workers were ≤ 25 years old and 53 (28.6) workers were more than 25 years old. Mean job duration of the subjects was 3.8 (± 3.3) years. Almost 80% workers had job experience of ≤ 5 years. Ninety five percent subjects were educated, while 5% were illiterate. One hundred & nine workers (58.9%) were unmarried. Majority of the workers (58.4%) were from grading section while 18.4% were from packing division. About 15% workers were engaged in peeling and ring cutting while rest of the workers were in mixed type of job.

So far as morbidity is concerned the workers were suffering from repeated injuries (49.7%), blanching during work (71.9%), recurrent musculoskeletal pain (61.1%) and recurrent (sneezing/coughing) respiratory irritation (14.6%). Almost 44% workers complained of headache during work while body ache and upper limb pain was reported by 4.9% and 22.2% subjects respectively (Table 1).

**Table 1:** Personal and occupational characteristics of workers

**Variables**	**Percentage (N = 185)**
***Age group***	
>25	28.6
***Job duration***	
>5	21.1
***Educational status***	
Educated (not illiterate)	95.1
***Marital status***	
Married	41.1
***Nature of Job***	
Grading	58.4
Peeling & Ring cutting	14.6
Packing	18.4
Mixed activity	8.6
***Work related morbidity***	
Frequent hand injury	49.7
Blanching of hand	71.9
Headache	43.8
Respiratory irritation (sneezing/coughing)	14.6
Body ache	4.9
Recurrent musculoskeletal pain	61.1
Recurrent upper limb pain	22.2

On univariate analysis some of the workers' characteristics were found to have significant effect on injury causation (Table 2). Age more than 25 years (RR; 1.56, 95%CI; 1.06–2.31), married status (RR; 1.5, 95%CI; 1.13–1.99), higher education level (RR; 0.62, 95%CI; 0.42–0.91), job of grading (RR; 1.96, 95%CI; 1.01–3.82) and packing (RR; 2.61, 95%CI; 1.32–5.14) as well as blanching (RR; 1.41, 95%CI; 1.06–1.86) and recurrent musculoskeletal pain (RR; 1.35, 95%CI; 1.02–1.79) has significant impact on injury causation while variables like job duration, headache, respiratory irritation did not show any significant impact. When multivariate analysis was done (Table 3) using logistic regression model to understand the effect of different worker characteristics on injury occurrence, it was observed that while analyzing with the first model (age, education level, marital status), marital status was found to have significant contribution on occupational injury occurrence. In the second model when morbidity parameters were added, it was observed that marital status lost its significance and only blanching of hand at work had significant effect. In the final/third model where all the variables were analyzed simultaneously, it was found that blanching of hand was the only work related morbidity, which had a significant impact on injury causation (OR; 2.30, 95%CI; 1.12–4.74). So far as the job characteristics are concerned, the act of grading (OR; 3.99, 95%CI; 1.41–11.27) and packing (OR; 5.68, 95%CI; 1.65–19.57) had significantly higher risk of contacting work related injuries.

**Table 2:** Association of worker characteristics on injury occurrence (univariate analysis)

**Variables**	**Injury (%)**	**P value**	**RR**	**95% CI**
***Age group***				
>25 (compared against ≤25)	64.2	0.013	1.56	1.06–2.31
***Job duration***				
>5 (compared against ≤5)	46.2	0.615	0.92	0.66–1.28
***Education level***				
Educated (compared against illiterate)	48.3	0.084	0.62	0.42–0.91
***Marital status***				
Married (compared against unmarried)	61.84	0.006	1.50	1.13–1.99
***Nature of Job***				
Grading	50.9	0.013	1.96	1.01–3.82
Peeling & Ring cutting	25.9		1	-
Packing	67.6		2.61	1.32–5.14
Mixed activity	43.4		1.69	0.72–3.93
**Blanching of hand**	54.9	0.025	1.41	1.06–1.86
**Headache**	51.9	0.610	1.08	0.81–1.44
**Recurrent respiratory irritation during work**	48.1	0.859	0.96	0.65–1.43
**Body ache**	33.3	0.313	0.74	0.46–1.20
**Recurrent musculoskeletal pain**	55.8	0.040	1.35	1.02–1.79
**Recurrent upper limb pain**	53.7	0.568	1.11	0.77–1.60

**Table 3:** Association of worker characteristics on injury occurrence (multivariate analysis)

**Variable**	**Regression Coefficient**	**Significance (p value)**	**Odds ratio**	**95% Confidence Interval**
Age	-0.0076	0.80	-	-
Education level	-1.2449	0.17	0.29	0.05–1.69
Marital status	0.6421	0.13	1.90	0.83–4.36
Job duration	-0.0533	0.35	-	-
Mixed job	1.0743	0.15	2.93	0.67–12.80
Grading	1.3829	0.009	3.99	1.41–11.27
Packing	1.7366	0.006	5.68	1.65–19.57
Blanching	0.8340	0.02	2.30	1.12–4.74
Musculoskeletal pain	0.5713	0.12	1.77	0.86–3.65
Upper limb pain	-0.1460	0.74	0.86	0.37–2.02

Main message:

Occupational hazards prevailing in a work environment can contribute significantly to injury occurrence also.

Policy implication:

Prevention of occupational hazards can protect workers from occupational injuries also.

## Discussion

Studies on fish processing workers have highlighted skin rashes, asthma and allergies as common work related symptoms [20]. Musculoskeletal problems have also been talked about [19,21]. Study conducted in Sweden on such workers showed that women workers are more susceptible to work related morbidities in comparison to their male counterparts despite superficially similar work [22]. So far as workplace injuries are concerned reports are there to show that injuries are higher in fish processing workers than non-exposed workers and women workers are more vulnerable than male workers (females: OR = 4.3; 95%CI = 3.0–5.9; males: OR = 1.8; 95%CI = 1.2–2.7) [23].

Though the studies conducted in fish processing industries have already highlighted that work related injury is a major problem area, hardly any study has explored the determinants. This present study has made an effort to identify the probable factors responsible for such work injuries so that this knowledge can ultimately help in prevention. On univariate analysis age group, marital status, education level, musculoskeletal pain, blanching of hand at work and nature of job showed significant contribution. But on multivariate analysis only blanching of hand and nature of job was found to have significance. Marital status showed significance in first model of multivariate analysis, but could sustain it at later stages. A peculiar pattern of such industries in India is that they almost exclusively employ women and most of these women (poor, less educated and migrated from different backward areas of the country) leave this job within 5 years of joining this job by the age of 25–26 yrs (mostly because of getting married). Those who stay here beyond 25–26 yrs are usually under mental tension either due to the social stigma of not getting married in time (in case of unmarried women) or due to the agony of staying away from the family (in case of married women). This may be the reason of such women having higher risk of injury. However, this increased risk was observed during univariate analysis and not in multivariate analysis, which indicates that this higher risk might have been observed due to the effect of other contributing factors. Though older women usually remain under mental tension (which may make them vulnerable to occupational injuries) they gather experience of job also with time. Naturally their on-job experience may contribute in protecting them from injuries also. For this reason we had an effort to see the effect of job duration also on injury causation but no significant contribution of experience could be found. Moreover, higher education level showed significant protective effect and mulculoskeletal pain showed significant contribution in univariate analysis. However, both of them lost their significance in multivariate analysis. This study has ultimately shown that apart from job pattern (grading and packing) work related morbidity (blanching of hand) has played significant role in the occurrence of work related injuries in fish processing workers. Taking special precaution during the job of grading and packing as well as alleviating (may be with the use of a personal protective equipment) the problem of blanching of hands during work may reduce the occurrence of work related injuries in a significant manner. This study has not only highlighted the problem of occupational injuries in Indian fish processing industries and the factors associated with such injuries but also has strengthened the findings of some recent studies [12-14] that have stated the role of poor work environment on occupational injury occurrence.

This study bears some limitations also. Inclusion of larger sample size (from different other parts of the country) in the study could not only have enabled us to explore the role of different personal and occupational characteristics (contributing variable for occupational injuries) in a greater detail but also could have made the results of this study more generalisable. Being a cross sectional study in nature, this study has suffered from the restriction of lack of temporality also.

This study eventually concludes that apart from nature of job of fish processing workers occupational hazards prevailing in the work environment contribute significantly to the occurrence of work related injuries and prevention of such occupational hazards may help in protecting workers from occupational injuries also.
